# Black-box assisted medical decisions: AI power vs. ethical physician care

**DOI:** 10.1007/s11019-023-10153-z

**Published:** 2023-06-05

**Authors:** Berman Chan

**Affiliations:** School of Philosophy and Sociology, Lanzhou University, Lanzhou, Gansu China

**Keywords:** Machine learning, Medical decisions, Opacity, Explainability, Physician care, Lithium

## Abstract

I raise an ethical problem with physicians using “black box” medical AI algorithms, arguing that its use would compromise proper patient care. Even if AI results are reliable, my contention is that without being able to explain medical decisions to patients, physicians’ use of black box AIs would erode the effective and respectful care they provide patients. In addition, I argue that physicians should use AI black boxes only for patients in dire straits, or when physicians use AI as a “co-pilot” (analogous to a spellchecker) but can independently confirm its accuracy. My argument will be further sharpened when, lastly, I give important attention to Alex John London’s objection that physicians already sometimes prescribe treatment, such as lithium drugs, even though neither researchers nor doctors can explain why the treatment works.

## Introduction

It is well known that machine learning algorithms hold great promise in using large data sets for pattern-recognition, prediction, and problem solving in numerous applications. The medical field, medical decision-making in particular, is just one of these important areas where machine learning algorithms can become very powerful tools. Such uses of artificial intelligence have yielded some exciting results in a wide range of medical applications, ranging from diagnosing eye diseases (Gulshan et al. 2016) or skin cancer from images (Esteva et al. 2017), to using clinical databases to predict the risk of suicide attempts (Walsh et al. 2017). The hope is that using AI algorithms will make medical diagnosis and treatment recommendation quicker and more accurate. Proponents may point out that AI will outperform human physicians, not only because of the sheer computing power AI possesses, but also because of the susceptibility of human clinicians to making diagnostic mistakes and succumbing to cognitive bias (Topol 2019). Note that machine learning is a subfield of artificial intelligence (AI) whose algorithms can learn from data or experience to improve performance. In this article I will be focusing on the (most powerful) *machine learning* techniques of AI.

Despite the great potential that AI has for revolutionizing health care, the computing prowess of some of the most powerful machine learning systems is accompanied by machine opacity. For example, deep learning algorithms are complex forms whose results can be opaque in the sense that even researchers would not understand the particular reasons why the algorithms have generated their results. When applied to medical decisions, then, physicians and researchers would not understand nor be able to explain why a particular diagnosis or recommendation is given. 1 In this sense, medical AI using these algorithms would be *black boxes.*

How exactly does AI machine learning work? As Alex John London (2019) explains, in the case of some deep learning applications, programmers construct a system architecture that can learn a mathematical model from a large set of data. This architecture contains many layers, and nested layers, of connected nodes that activate when they detect particular features of input data. The system learns, in most cases, when data from known classifications (e.g., images from retinas that have, or lack, diabetic retinopathy) are inputted into the system during a “training” phase. During training, weights on the nodes in the network are adjusted accordingly, to construct a mathematical model that most accurately maps certain inputs (e.g., images of retinas or patient medical records) to the right output classification (e.g., retinopathy or not, or some medical event or not). After the training phase, the reliability and precision of the system can be tested by applying it to a second set of data whose classification is also known, and comparing the output classifications generated by the system with the known classifications. Such deep learning systems can be trained using large data sets containing millions of inputs, and the resulting predictions can be very accurate. But London cautions:

Despite this accuracy, deep learning systems can be black boxes. Although their designers understand the architecture of these systems and the process by which they generate the models they use for classification, the models themselves can be inscrutable to humans. Even when techniques are used to identify features or a set of features to which a model gives significant weight in evaluating a particular case, the relationships between those features and the output classification can be both indirect and fragile. A small permutation in a seemingly unrelated aspect of the data can result in a significantly different weighting of features. Moreover, different initial settings can result in the construction of different models. (London 2019, 10)

So, since the models (mapping certain inputs to output classifications) can be inscrutable to humans, the reasons for AI-generated decisions can also be inscrutable. Thus, in this sense, the AI algorithms generating medical decisions can be black boxes, and these decisions can be said to be “black box decisions”.

This machine opacity has been discussed in the literature with regards to ways of mitigating that opacity to increase our trust in the AI’s accuracy (Grote 2021; Durán and Jongsma 2021; Ploug and Holm 2020). Machine opacity has also been discussed as introducing the possibility of medical paternalism (McDougall 2019; Grote and Berens 2020) or compromising on a variety of important values such as fairness, the authority of physicians (e.g. Grote and Berens 2020), or patients’ data-privacy (Ploug and Holm 2020). In this article, I will instead make the case that the use of black box AIs would *compromise physician care for patients* when it comes to diagnosis and treatment selection. Even though I will agree about the risk of medical paternalism, the thrust of my argument will be that care for patients (in diagnosis and treatment selection) would also be compromised in other specific ways, especially having to do with efficacy and respect for patient dignity (in addition to patient autonomy). Proper physician care embodying these values, I will contend, must be able to explain diagnoses and treatment recommendations, and physicians using AI medical black-boxes would erode this morally-obligated physician care—it would at least introduce a significant trade-off between it and AI power. My argument will be specifically against physicians’ *straightforward* uses of black box medical AI (i.e. uses without understanding the reasons for the medical decisions, and uses in general but not special cases). For I will also argue that physicians should use medical AI only (i) in special cases, that is, for patients in dire straits where the potential benefits significantly outweigh drawbacks with respect to care, or (ii) when physicians use AI as a “co-pilot” (analogous to a spellchecker) but can independently confirm the accuracy of the AI’s decision. My argument will be further sharpened when, lastly, I give important attention to the interesting objection that physicians already prescribe some treatments, such as lithium drugs, even though they do not understand why they are effective.

## The case for physicians to avoid the straightforward use of black box AI

Why would using medical AI compromise a physician’s care for patients? Why does good physician care entail the ability to explain their medical decisions? My argument will draw upon the American Medical Association (AMA)’s code of medical ethics, although every principle appealed to can also be found in other major medical codes. I choose the AMA’s code as it is quite influential, and was inspired by Thomas Percival’s 1803 classic *Medical Ethics* (see the 2014 reprint)—the first medical ethics code in modern history.^2^ I offer a simple argument for my position, starting with the plausible principles that doctors have a moral responsibility to provide their patients with medical care that is effective (American Medical Association, “Quality”) and respects their dignity (American Medical Association, “Patient Rights”, a) and autonomy (American Medical Association, “Patient Rights”, b-d).^3^ The lynchpin of my argument is this: Treating patients according to these values requires being able to explain diagnoses and treatment recommendations. On the other hand, if physicians used black box AI to select diagnoses or treatment recommendations, it would be the AI providing the diagnoses or treatment recommendations, and physicians would not be able to understand nor explain these decisions. Nor can AI explain them to patients, because of machine opacity.^4^ So, doctors should avoid straightforward uses of medical AI. Granted, physicians should also have an eye towards new technologies that can improve patient outcomes (American Medical Association, “Quality”, c). So, I contend that physicians are morally permitted to use AI under two sorts of scenarios that I will discuss in Sect. 3. However, as their black box nature would compromise doctors’ ability to explain results and thereby adequately treat patients in accordance with the values of effectiveness, dignity, and autonomy, medical AIs would in those respects take away from good patient care. Physicians, thus, have a moral responsibility to avoid *straightforwardly* using medical AI in their care of patients (whether in diagnosis, recommending treatment, or both).

The lynchpin of the above argument is what I will call the “Core Claim”.

Core Claim: Treating patients with effectiveness and respect for their dignity and autonomy requires being able to explain medical diagnosis or treatment recommendation.

Why should we accept the Core Claim? Let me offer two reasons in a cumulative case to support it.

The first reason to accept the Core Claim is that communicating a diagnosis is a delicate matter. Especially if it is an upsetting diagnosis, I argue it should be communicated by a physician *who is able to explain the diagnosis*, if requested. (And so, even if a human physician working in tandem with medical AI took the AI-generated diagnosis and communicated it to the patient, patient care would be eroded if the physician cannot explain the diagnosis.) Why? I contend that the physician being able to explain the diagnosis can stave off denial on the part of the patient and increase the chances of effective treatment. Granted, some patients may prefer denying an upsetting diagnosis as a way of coping (Babrow et al. 2000). However, for those who initially deny it but only because of lack of information, the physician’s ability to explain the diagnosis can help patients dispel doubt that would otherwise stand in the way of effective treatment (Sharf et al. 2005, 644). Second, the physician being able to explain to the patient how the upsetting diagnosis was arrived upon could give them an element of personal dignity, because the patient would at least gain some sense of understanding of why they received it.^5^ Also, even in cases involving non-upsetting diagnoses, what some patients desire is to ask questions and for them to be dignified and respected with an informative answer. A team of researchers in physician-communication put it this way: “[Some patients] want their physicians to respect them as important, active partners in their care” (Lee et al. 2002, 478). They continue, “Patients have more faith in a physician who admits to not knowing the answer to a question and promises to investigate it, than in a physician who dismisses the question out of hand” (479). As one stem cell transplant survivor notes, “Not everyone wants lots of info. We did, and when we didn’t get it, we felt like they were deliberately hiding things from us. Our docs treated us like we were intelligent, but didn’t make fun of us when we were really dumb!” (Lee et al. 2002, 479). While doctors probably cannot give answers to absolutely all a patient’s questions, providing them informative answers to some of their important questions is crucial to treating them with dignity. It is likely for this reason that the American Medical Association’s code of ethics opinion states that patients have a right to ask questions about their health status (and recommended treatment) and to have them answered by their physician (“Patient Rights”, a.2).^6^ But black box AI would prevent even doctors from understanding the reasons for the diagnosis, not to mention patients. Thus, using medical AIs for diagnosis compromises patient care.

There is a second reason that treating patients properly requires being able to explain medical decisions. This reason has to do with offering treatment recommendations. In essence, rather than merely communicating the AI’s recommendation, being able to him/herself explain the recommended treatment is necessary for the doctor to achieve two objectives: (a) to properly advise the patient in selecting a treatment option, and (b) to motivate the patient to persevere with the chosen treatment. Doing these two things are prerequisites for treating patients effectively and respectfully.

Beginning with the first objective, advising the patient regarding different treatment options is part and parcel of the physician’s ethical duty to provide information so that the patient can make an informed choice of treatment (American Medical Association, “Patient Rights”, a. 2). This potentially gives patients some sense of understanding, acceptance, and control of the situation, thereby affording them with dignity and autonomy. However, in order to actually advise the patient regarding treatment options, the physician would of course need to grasp the issues surrounding the options, and understand the reasons for recommending a particular option “based on the physician’s objective professional judgment”, as the AMA code of medical ethics puts it (“Patient Rights”, a. 2). If, instead, the physician simply used a black box AI to decide, the doctor would be unable to meaningfully explain why the different treatment plans are on the table (and perhaps why some others are not). This would rob the patient of the understanding of the eventual treatment plan. This would also take away some significant element of choice, at least informed choice, since the doctor would not be able to substantively advise the patient which treatment to choose (Grote and Berens 2020, 208 and Bjerring and Busch 2021, 363). McDougall (2019) has also raised an interesting point that medical AI would assume certain priorities that guide the ranking of the different treatments it recommends, perhaps prioritizing maximizing lifespan at the cost of more suffering, for example. This risks compromising the patient’s autonomy in the decision-making process. I think, however, that once the physician reads the list of treatment options recommended by the AI, the physician would be able to tell the patients roughly what side effects (or drawbacks) come along with each treatment.

But even if the physician can tell the patient what drawbacks and benefits come with a treatment, without understanding the specific rationale leading the AI to recommend it, and the rationale behind the AI’s treatment prediction (i.e. % confidence of success), it may be difficult for the patient to choose between treatments – the patient might not be able to give *informed* consent to their choice in a way that balances their personal priorities. So, the patient would be robbed of some measure of patient autonomy (because they would not be able to make an informed decision) as well as some degree of dignity in this situation (because they would lack a sense of understanding and thereby lack some degree of acceptance of the eventual decision). Now, it could be objected, though, that medical AIs in the future can come equipped with interpretability tools that can disclose some relevant information (in the decision process) to the patient, such as expected lifespan and expected suffering. However, as Grote and Berens (2020, 208) ask, what information is essential to tell the patient and what others can be withheld? I agree this is a thorny issue, and perhaps Grote and Berens are even hinting that the problem is further compounded by the fact different patients might need different information. For example, for serious conditions, many patients would want to know the expected lifespan and expected pain/discomfort from some treatment, while others information such as the probability of avoiding brain fog, the odds of continuing to function sexually, etc. Further, what is experienced as suffering, and how much, can depend heavily on the individual.

Not only is a doctor being able to explain treatment options necessary so that, as just shown, the patient can be properly advised in selecting among the various options, but as articulated by objective (b) above, being able to explain them is also sometimes necessary for the doctor to motivate the patient to persevere with the chosen treatment. The physician’s ability to explain why a treatment was recommended is especially important when there may be significant side effects from treatment. Whether the treatment plan is something like chemotherapy, taking medications, or whatever, side effects often become obstacles to the patient persevering in that treatment.^7^ This is where it is effective that the physician is able to explain and justify the choice of treatment, rather than just say that one should trust because AI knows best. Side effect or not, there is evidence that answering all of a patient’s questions is a predictor of patients’ adherence to treatment (DiMatteo et al. 1993). Also, where side effects exist, precisely because they may be significant, the physician may want to suggest a few things (or prescribe something) to mitigate the side effects. But, so as not to undermine the treatment, this may sometimes require the doctor to understand, roughly, how the treatment works to cure the patient’s ailment (or at least understand the factors weighted heavily^8^ in choosing that treatment for the patient). So, the doctor being able to explain a treatment recommendation is sometimes needed to encourage a patient’s adherence to treatment.

Thus, for the above reasons, we should accept the Core Claim, which asserts that treating patients with effectiveness and respecting their dignity and autonomy requires being able to explain medical decisions. This claim was a crucial premise in the argument I offered at the beginning of Sect. 2, and on the strength of that argument, we should conclude that physicians have a moral responsibility to give this medical care to their patients, and to avoid straightforwardly using medical AI black boxes.

## Permissible uses of black box medical AI

There are, however, two conditions under which a physician would be morally permitted to use black box medical AIs. The first is associated with cases where the medical algorithm generates a diagnosis or treatment plan, but once these results are presented the physician can independently confirm their accuracy (call this the “co-pilot” use of medical AI). After all, diagnostic errors are quite common in health care. The (US) National Academy of Sciences, Engineering, and Medicine estimates that 5% of all US adults who seek annual outpatient care experience a diagnostic error. In addition, they report that diagnostic errors contribute to about 10% of patient deaths (2015). In contrast, machine learning algorithms can use and process large sets of data in a short amount of time, and are less prone to making errors. A good comparison with the “co-pilot” use of medical AI is a writer’s use of spelling and grammar checkers—even though a writer may miss a better way of writing some sentence (e.g., active rather than passive voice) or miss a spelling or grammatical error, once the checker points out an error or offers a suggestion, the writer can independently confirm the error or the superiority of the suggestion. In an analogous way, after making an initial decision about a diagnosis or treatment, a doctor might then consult a medical AI, which might point out some diagnosis or treatment that the physician did not think of (or perhaps even rule out certain diagnoses or treatments). But perhaps once the medical AI has brought these results to the physician’s attention, the doctor can independently see the accuracy of the AI’s suggestion even though the AI does not provide its reasoning (since it is a black box). That is, the doctor can independently confirm its accuracy by way of the his/her own professional experience, knowledge of clinical studies, or by recollecting some other relevant information about the patient, etc.^9^ However, if the physician cannot independently confirm the medical AI’s conclusion, then for all the reasons stated in Sect. 2, medical AIs should not be used by the physician.

Granted, the co-pilot use comes with the risk that physicians might become biased towards agreeing with the AI’s decision, assuming its accuracy is known to be reasonably high (Grote and Berens 2020, 208). Or, instead the physician might remain steadfast in their original decision in spite of the AI’s decision, but should the human’s diagnosis turn out to be wrong or treatment recommendation ineffective, the doctor might be accused of being professionally irresponsible for not heeding medical AI. In response to the first case, perhaps novice doctors would likely be most susceptible to bias in favour of the AI’s decision (Grote 2021), and so one might counsel that they refrain from using medical AI until they become more experienced. In response to the second case, the physician might consult a human medical peer to reduce cognitive bias,^10^ and if the first physician thereby comes around to agree with the AI’s decision (with understanding), this case would then collapse into the co-pilot use of AI. If instead the physician still does not agree with the AI’s decision, then I think it ethically and professionally justifiable for the doctor to continue advising according to their own medical judgment instead of the AI’s, firstly because of their ethical duty to provide their patient guidance according to their own objective professional judgment (American Medical Association, “Patient Rights”, b), and secondly because of the significant benefits to patient care already argued for in Sect. 2. Another reasonable option is for the physician to let the patient know that the AI disagrees with the physician’s decision, answer the patient’s questions, explain that medical AIs have an opaque aspect, and then let the patient or patient advocate decide. All in all, I do acknowledge that the introduction of AI into the medical field does simultaneously introduce a sort of medical peer alongside human physicians, and where there exists such peer disagreement, many important thorny epistemic and ethical questions are raised (Grote and Berens 2020). Without wading into all these issues in this article, though I have just made some remarks on the matter, my emphasis in this article is the claim that it is ethically permissibility for a physician to use AI as co-pilot if the former can independently understand and confirm the accuracy of the latter’s decision.

I argue that another kind of condition under which a physician would be morally permitted to use black box medical AIs are cases where the patient’s situation or prognosis is so poor that there would be little to lose by sacrificing explainability in patient care. Presumably in these cases, the physician’s diagnosis has lacked explanatory power, and/or the associated treatment has not been effective. Medically, the patient is in dire straits or would suffer a lot, and so the potential benefits of using medical AIs would significantly outweigh whatever erosion of physician care for the patient that may occur from opacity. Now, the patient would have to give informed consent, and the case would have to satisfy some other reasonable criteria. These criteria, in many ways, could mirror those justifying the use of experimental therapies (Bunnik et al. 2018), which are also sometimes advocated for patients in dire straits.

## Objection

I think the best objection to my argument that physicians should avoid straightforward uses of black box medical AIs is the point that physicians already sometimes give treatment recommendations even though they do not understand why the treatments are effective. So, in this sense “black boxes” are already commonly used in medical practice, and thus medical AIs should not automatically be avoided simply for this reason. Alex John London makes this point, arguing that medical science is more complex than structural engineering and thus not as completed of a science. So, we should not expect that physicians can always explain why medical treatments are effective. London points out that physicians regularly prescribed aspirin for nearly a century without understanding the mechanism by which it worked. Lithium has been used as a mood stabilizer to treat mental illness for many decades, but to this day researchers cannot explain why it works (London 2019, 17). So, the fact that medical AIs are black boxes should be no reason to avoid using them in medicine. Furthermore, the objector may continue to press that since a black box medical AI uses large sets of data to give a treatment recommendation, its recommendation would be based on something at least as reliable as the supporting data in lithium’s randomized clinical trials. Hence, the objector may argue that once physicians are aware of the treatment idea proposed by medical AI, physicians can themselves choose to recommend that treatment on the basis of its being supported by data.

However, the comparison with lithium’s clinical trials here is inapt. The reason is that doctors prescribing lithium still understand the basic factors about their patient and lithium that weight heavily in the decision to prescribe it. When deliberating about prescribing lithium, in addition to knowing that their patient suffers from mood instability and other facts about the patient, they can also recall what they have heard of clinical trials (or, more likely, expert summaries) of lithium. They can recall (or look up again) the most basic and relevant factors in the trials that would lead them to prescribe it to their particular patient, simple factors perhaps such as age or gender demographics of the trial subjects, as well as treatment success rates (which supporting trial data could even be looked up if needed). So, they would understand the basic factors leading them to prescribe lithium to their particular patient. In contrast, when physicians use black box AI, while the AI could presumably indicate the probability that the treatment will be effective (though support for the figure would be opaque), physicians would have very little information beyond that to give them understanding of the particular reasons for recommending that treatment, e.g., what factors about their patient (and perhaps recommended treatment) are weighted heavily in the decision to recommend the treatment. So, while physicians would not know the weighted factors in the AI decision, doctors would know them in their decision to prescribe lithium. While the weighted factors by themselves likely do not provide an adequate explanation of the reason for the decision, they could be a first step towards one.^11^ But these weights, contained in the deep learning model, can be inscrutable even to researchers.^12^ Even if medical AI indicated to doctors information such as the ethnic and age demographics associated with the data sets that the AI used (e.g., from clinical trials), whether or not those factors were *weighted features in the model* would be inscrutable. Similarly, even if the AI was programmed to take all the patient’s demographic details into consideration, AI could not tell the physician *which* of these details were heavily weighted.^13^ Hence, these considerations serve to show that the comparison with lithium prescription is inapt.^14^ Thus, the permissible prescription of lithium cannot be used to justify physicians’ straightforward use of medical AI to recommend treatment.

Now, at this point, a proponent of black box medical AIs might counter that it is precisely the fact that physicians often know of clinical trials for various therapies, that once the AI recommends a therapy, then the doctor *is* aware of (or can search online for) the most basic and relevant factors in the trial that would lead them to knowledgeably prescribe it to their particular patient. Thus, so the proponent argues, even while using black box AIs, by considering clinical trials corresponding to the treatment that the algorithm recommends, the physician would then understand the basic factors for recommending that treatment. However, in response, I think the reader can see that this sort of case would be an instance of what I called the “co-pilot” use of medical AI. That is, the AI is used only to, say, bring to the physician’s attention treatments that they did not consider, but after the AIs result is given to the physician, the latter can independently confirm the accuracy of its decision. I argued above in favour of the co-pilot use.

However, the question under discussion in this article includes cases going beyond merely the co-pilot use. For there will be many cases where the black box AI will recommend treatments whose associated clinical trials would not report efficacy for treating the particular illness affecting that patient (if instead they did indicate efficacy in treating that particular illness, then once the doctor learns this and recommends the treatment, this would be using AI as a co-pilot). This is an important kind of scenario, because it is precisely the greater ability of medical AIs to use enormous data sets to find solutions beyond what a human physician could already know of (or even be able to search for) that makes using black box AIs attractive and advantageous. AIs are not limited to merely the results and information contained in reports of clinical trials (or even meta-analyses of them). Instead, AI can potentially use bits and pieces of the data sets connected with clinical trials (large numbers of them), and in conjunction with other medical and research data, generate treatment recommendations using complex associationist algorithms. But then, as a result, for all non-co-pilot uses, AI will recommend a treatment and when the doctor looks into published trials of that treatment, if they exist they would not discuss efficacy treating the condition(s) ailing the physician’s patient; they would discuss efficacy treating some other ailment(s). So, even if, say, the physician was encouraged that the trials included subjects having demographic features that were similar to the patient’s, this would be irrelevant to helping the physician understand the reasons (and weighted factors) that this treatment was recommended by AI to treat their patient’s own condition.

Even if their patient’s specific ailment is close to the ones that trials report efficacy for, either it is so close that the physician can judge that the treatment would likely be effective (and this would thus be a type of co-pilot use of AI), or not so close, in which case the physician would not be confident enough to recommend it. In the latter case the human physician would understand neither the reasons nor even the particular weighted factors leading to its selection by AI, and so using medical AI would negatively affect patient care in the ways explained in this article. Thus, the straightforward use of medical AIs is neither defensible by appeal to lithium prescription nor by appeal, relatedly, to the availability of clinical trials for the treatment recommended by black box AI.

Consequently, we do have a principled basis for rejecting physicians’ straightforward use of black box medical AIs for generating diagnoses or treatment recommendations, while still allowing physicians to prescribe drugs like lithium. Thus, aside from the two permissible uses of black box medical AI discussed above, in view of their moral responsibility towards patients, physicians should avoid using it, to preserve the level of care for patients ensured by a doctor who is able to explain their medical decisions.

## Footnotes

1 Following Mittelstadt et al., I take explanation (of a computed decision) to need to be contrastive, selective, and social, and to include methods allowing for the justification of the decision to be “debated and contested” (Mittelstadt et al. 2019, 286). I hold that this is also what is needed for explanation in the medical context, to render it no longer opaque. While I follow Mittelstadt et al.’s conception, my argument and position could turn out to be compatible with different conceptions of explanation. Regarding the importance of explanation in AI as *contrastive*, see also Miller, T. (arXiv), and Holzinger, A. et al. (2022, 267). For the importance of *contestability*, see also Ploug, T. & Holm, S. (2020).

2 See Baker and Emanuel (2000) for the historical influence of the AMA’s code in the US and the English-speaking world, as well as its worldwide impact via the AMA’s influence upon the Nuremberg Code to protect the human rights of test subjects, e.g., requiring the informed consent of human experimental subjects (Baker and Emanuel 2000 S14-5).

3 The AMA code contains numerous ethical principles (“Ethics Opinions”) governing the conduct of physicians and patients, but I will be singling out these above and a few other principles because they are the ones most relevant to our discussion of the physician’s use of AI. Other principles articulated in the AMA’s code that may be relevant either overlap with the ones I single out, or are not reasonably relevant, such as ones governing terminating a patient-physician relationship or treating oneself or family.

4 Black box algorithms being unable to explain decisions to patients could form the basis of a similar argument against deploying medical AIs without an accompanying human physician. However, my article focuses on the physician-accompanied scenario. The argument against the unaccompanied use of medical AIs, incidentally, may need to support why AIs (or healthcare systems – not physicians–) are morally obligated to treat patients according to the values discussed (e.g., effectiveness, dignity, autonomy).

5 As for what dignity means, one might use Suzy Killmister’s concept of “dignity-as-bearing” (Killmister 2022), where S’s having dignity involves S choosing certain actions or modes of presentation (while avoiding others), and S’s having dignity is also vulnerable to the actions or omissions of others (e.g., that put S in humiliating situations vs. where S retained some sense of honour, etc.).

6 Ploug & Holm (2020, 2) argue that, even when medical AI is used, it is a matter of individual rights that a patient should be able to ask such questions and to be answered. They contend further that AI explainability is effective contestability. Their argument is framed from the perspective of the patient, whereas mine primarily from that of the physician (i.e. physician’s ethical responsibility). However, I obviously agree that patients have a right to having their important questions answered by their physician, and concur that explanation must include contestability (although, following Mittelstadt et al. 2019], there is more to it – i.e. it must be contrastive. See my footnote 1).

7 Sometimes, say for some mental illness, medications are meant to be taken regularly even if the patient is feeling good. But the side effects are significant, and so they may stop taking the medicine. Chemotherapy obviously comes with severe side effects.

8 More discussion about weighted factors will appear in Sect. 4 below.

9 The physician might also consult a colleague regarding the AI’s result, and this discussion may help the first doctor understand and become able to confirm the AI’s decision. (I touch on an instance of this possibility in the following paragraph as well.)

10 Cognitive bias is a tendency to allow one’s judgement to be unjustifiably or inordinately swayed by pre-existing beliefs. Please see Doherty and Carroll (2020) for a helpful description of four common forms of cognitive bias in medical practice.

11 Amann et al. (2020, 5) also concur that explanation requires reference to the weighted features. But I think more is needed; recall my footnote 1 citing Mittelstadt et al.’s (2019) conception of explanation that is contrastive and allows for discussion (contestability) of the decision’s justification. Yet, weighted features (in a decision) could figure into such explanations, I argue, when a relevant alternative data point that contrasts with a weighted feature would return a different decision. For instance, with regards to the human physician’s decision to prescribe lithium for mood stability, the weighted feature of patient age (or ethnicity) is amenable to such contrastive explanation by considering that, e.g., *if my patient’s age or ethnicity were different such that it was very unrepresented by the subjects of the clinical trials, then I would not prescribe lithium.* In addition, such contrastive explanation (provided by a physician) using weighted features does also allow for discussion (contestability) of the decision’s justification, as physicians can field questions about the weighted features and use contrastive reasoning to assuage the interlocuter’s contestation.

12 Medical AIs sometimes come with interpretability tools that can identify weighted factors, and Aman et al. (somewhat optimistically perhaps about their accuracy) enjoin clinicians to use the tools (2020, 7). However, as pointed out earlier, the relationship between weighted factors and the AI’s decision can be fragile and tenuous (London 2019, 17). All this seems surprising of weighted features, especially ones identified through post hoc techniques (that might pick out key features leading to the decision, after the fact). However, one possible reason is that the post hoc interpretation often does not represent precisely how the model works (Lipton 2018, 40). However, consider sufficiently big breakthroughs in interpretability, e.g., tools providing contrastive explanation for decisions, amenable to contestability (Mittelstadt et al. 2019). Then, some of my arguments may no longer apply. But to the degree there is effective interpretability, to that degree the AI is not black box. Still, even with contrastive explanation, the problem mentioned earlier (Sect. 2) might remain about different patients needing different information from the interpretability tool for informed consent (e.g., regarding brain fog, sexual function, other information).

13 What if the algorithm could give physicians the option of *forcing* it to weight certain demographic details? Problem: This might seriously hamstring the accuracy of the algorithm’s result, because the associationist algorithm would normally find its own weighted factors and features. Zachary Lipton gives a similar warning that forcing machine learning models to be transparent would degrade its predictive power and clash with the goal of improving medical outcomes (Lipton 2018, 43).

14 A similar comparison-based objection is found in Durán and Jongsma’s comparison with doctors’ use of MRI machines, whose inner workings are black boxes to physicians (Durán and Jongsma 2021, 333). My reply: But the physician knows the specific MRI data (e.g., images of abdominal tumours) that they decide to weight heavily in their diagnosis.

## References

[CR1] Amann J, Alessandro B, Vayena E, Frey D, Madai V (2020). Explainability for artificial intelligence in healthcare: a multidisciplinary perspective. BMC Medical Informatics and Decision Making.

[CR2] American Medical Association, “Patient Rights”, Code of Medical Ethics Opinion 1.1.3, Available: https://www.ama-assn.org/delivering-care/ethics/patient-rights [Accessed July 18, 2022].

[CR3] American Medical Association, “Quality”, Code of Medical Ethics Opinion 1.1.6, Available: https://www.ama-assn.org/delivering-care/ethics/quality [Accessed July 18, 2022].

[CR4] Babrow, A. S., S. C. Hines, and C. R. Kasch. 2000. Managing uncertainty in illness explanation: an application of problematic integration theory. In *Explaining Illness: Research, Theory, and Strategies, Whaley BB (ed.)*, Mahwah, NJ; 41–67. Lawrence Erlbaum Associates.

[CR5] Baker R, Emanuel L (2000). The efficacy of Professional Ethics: the AMA Code of Ethics in historical and current perspective. The Hastings Center Report.

[CR6] Bjerring JC, Busch J (2021). Artificial Intelligence and patient-centered decision-making. Philosophy of Technology.

[CR7] Bunnik EM, Aarts N, van de Vathorst S (2018). Little to lose and no other options: ethical issues in efforts to facilitate expanded access to investigational drugs. Health Policy.

[CR8] DiMatteo MR, Sherbourne CD, Hays RD, Ordway L, Kravitz RL, McGlynn EA (1993). Physicians’ characteristics influence patients’ adherence to medical treatment: results from the Medical Outcomes Study. Health Psychology.

[CR9] Doherty T, Carroll A (2020). Believing in overcoming cognitive biases. AMA Journal of Ethics.

[CR10] Durán JM, Jongsma KR (2021). Who is afraid of black box algorithms? On the epistemological and ethical basis of trust in medical AI. Journal of Medical Ethics.

[CR11] Esteva A, Kuprel B, Novoa RA, Ko J, Swetter SM, Blau HM, Thrun S (2017). Dermatologist-level classification of skin cancer with deep neural networks. Nature.

[CR12] Grote T (2021). Trustworthy medical AI systems need to know when they don’t know. Journal of Medical Ethics.

[CR13] Grote T, Berens P (2020). On the ethics of algorithmic decision-making in healthcare. Journal of medical ethics.

[CR14] Gulshan V, Peng L, Coram M (2016). Development and validation of a deep learning algorithm for detection of diabetic retinopathy in retinal fundus photographs. Journal of the American Medical Association.

[CR15] Holzinger A, Dehmer M, Emmert-Streib F (2022). Information fusion as an integrative cross-cutting enabler to achieve robust, explainable, and trustworthy medical artificial intelligence. Information Fusion.

[CR16] Killmister, S. 2022. Autonomy and Dignity. In Ben Colburn (ed.), *The Routledge Handbook of Autonomy*. 167–177.

[CR17] Lee SJ, Back AL, Block SD, Stewart SK (2002). Enhancing physician-patient communication. ASH Education Program Book.

[CR18] Lipton ZC (2018). The mythos of model interpretability. Communications of the ACM.

[CR19] London AJ (2019). Artificial intelligence and black-box medical decisions: accuracy versus explainability. Hastings Center Report.

[CR20] McDougall RJ (2019). Computer knows best? The need for value-flexibility in medical AI. Journal of Medical Ethics.

[CR21] Miller, T. Contrastive explanation: a structural-model approach. arXiv preprint arXiv:1811.03163.

[CR22] Mittelstadt, B., C. Russell, and S. Wachter. 2019. Explaining explanations in AI. *Proceedings of the Conference on fairness, accountability, and transparency*: 279–288.

[CR23] National Academies of Sciences, Engineering, and Medicine. Improving diagnosis in health care. National Academies Press, 2015.

[CR24] Percival T (2014). Medical Ethics: or, a code of Institutes and Precepts, adapted to the Professional Conduct of Physicians and Surgeons.

[CR25] Ploug T, Holm S (2020). The four dimensions of contestable AI diagnostics - a patient-centric approach to explainable AI. Artificial Intelligence in Medicine.

[CR26] Sharf BF, Stelljes LA, Gordon HS (2005). ‘A little bitty spot and I’m a big man’: patients’ perspectives on refusing diagnosis or treatment for lung cancer. Psycho-Oncology.

[CR27] Topol EJ (2019). High-performance medicine: the convergence of human and artificial intelligence. Nature medicine.

[CR28] Walsh CG, Ribeiro JD, Franklin JC (2017). Predicting risk of suicide attempts over time through machine learning. Clinical Psychological Science.

